# Addressing the growing opioid and heroin abuse epidemic: a call for medical school curricula

**DOI:** 10.1080/10872981.2018.1466574

**Published:** 2018-04-30

**Authors:** Madison C. Ratycz, Thomas J. Papadimos, Allison A. Vanderbilt

**Affiliations:** a College of Medicine and Life Sciences, University of Toledo, Toledo, OH, USA; b Simulation Center Department of Anesthesiology, College of Medicine and the Life Sciences, University of Toledo, Toledo, OH, USA; c Curriculum Evaluation and Innovation, Family Medicine, College of Medicine and Life Sciences, University of Toledo, Toledo, OH, USA

**Keywords:** Medical education, LCME, opioid, heroin, curriculum

## Abstract

Substance abuse is a growing public health concern in the USA (US), especially now that the US faces a national drug overdose epidemic. Over the past decade, the number of drug overdose deaths has rapidly grown, largely driven by increases in prescription opioid-related overdoses. In recent years, increased heroin and illicitly manufactured fentanyl overdoses have substantially contributed to the rise of overdose deaths. Given the role of physicians in interacting with patients who are at risk for or currently abusing opioids and heroin, it is essential that physicians are aware of this issue and know how to respond. Unfortunately, medical school curricula do not devote substantial time to addiction education and many physicians lack knowledge regarding assessment and management of opioid addiction. While some schools have modified curricula to include content related to opioid prescription techniques and pain management, an added emphasis about the growing role of heroin and fentanyl is needed to adequately address the epidemic. By adapting curricula to address the rising opioid and heroin epidemic, medical schools have the potential to ensure that our future physicians can effectively recognize the signs, symptoms, and risks of opioid/heroin abuse and improve patient outcomes. This article proposes ways to include heroin and fentanyl education into medical school curricula and highlights the potential of simulation-based medical education to enable students to develop the skillset and emotional intelligence necessary to work with patients struggling with opioid and heroin addiction. This will result in future doctors who are better prepared to both prevent and recognize opioid and heroin addiction in patients, an important step in helping reduce the number of addicted patients and address the drug overdose epidemic.

Substance abuse is a major public health issue in America, especially given that the USA (US) is currently facing a national epidemic of overdose deaths, with more people dying from overdoses than ever recorded before [1]. From 1999 to 2016, drug overdose deaths more than tripled [2], making overdose the leading cause of accidental death in the US [3]. The increase in opioid abuse is one of the driving factors of this epidemic; currently more than half of overdoses in the US involve the use of opioids [4]. In 2016 in particular, there were over 63,600 drug overdose deaths, and opioids played a role in 42,249 of these [2,5].

In recent years, however, heroin and synthetic opioids in particular have contributed to the increased overdose rates [1,4]. In 2015, approximately 591,000 people had a heroin-use disorder in particular [6]. A heroin-use disorder is defined as an individual who uses heroin for potential drug-related euphoria, abuses heroin, or is physically dependent on heroin and struggles to stop using it. From 2010 to 2016, the number of heroin-related overdose deaths showed a fivefold increase and death rates for heroin increased by 20% from 2015 to 2016 alone [7]. One factor that may account for the sharp increase in the number of heroin users is the potential association between prescription opioid misuse and heroin use [7], as roughly 80% of heroin users report first abusing prescription opioids [8]. Possible reasons for this association include the increased availability and lower cost of heroin, making it easier to obtain [7, 9–12]. Similar to increased rates of heroin overdose, rates of overdoses from synthetic opioids (excluding methadone) increased by 88% between 2013 and 2016 [2]. This significant increase seems to be primarily the result of illicitly manufactured fentanyl [13,14], which is commonly mixed into batches of heroin [15]. This is concerning given that fentanyl is 50–100 times more potent than morphine [16].

The issue of heroin and fentanyl abuse is a national public health crisis that needs to be addressed in order to counteract the devastating effects it has across the country, ranging from high numbers of overdose deaths and increasing incidences of medical conditions associated with intravenous drug use, such as HIV and Hepatitis C [17,18]. Successfully addressing the opioid and heroin epidemic requires a collaborative approach from law enforcement, public health, and social service agencies, as well as physicians [19,20]. Physicians serve a vital role within the opioid and heroin epidemic as it relates to (1) education (2), prescription of medication, and (3) screening and referrals for treatment of addiction. With regards to education, physicians can provide educational materials along with verbal information for patients and their families about the risks related to developing opioid addiction. For those patients who are at high risk to develop the addiction from an opioid to heroin transition, additional educational conversations should occur between the physician, patients, and families. When physicians take into consideration the use of prescription of opioids for pain management, it is essential to consider if their patient is at risk for drug-seeking behavior, the differential diagnoses, and potential misuse of medication. Finally, when physicians screen for addiction, the patient history is one of the most critical aspects for identifying addictive behavior.

Given the role of physicians in dealing with patients at risk for abuse or patients who are abusing (opioids/heroin), it is of utmost importance that physicians are aware of this issue and know how to respond appropriately. Many physicians lack knowledge regarding the identification, assessment, and treatment of opioid addiction [21,22]. Increased awareness and education among physicians will enable them to identify patients at risk for substance abuse and overdose and manage patient care more effectively. Unfortunately, a report from 2012 indicated that medical school curricula do not adequately cover or spend substantial time covering addiction medicine and that most doctors fail to identify or treat patients with substance abuse problems [23–25].

Recognizing the growing importance of medical school curricula in effectively addressing the national drug overdose issue, the Association of American Medical Colleges created a statement that 74 medical schools signed in order to demonstrate their willingness toward better incorporating opioid-related topics in their training of medical students [26,27]. An *AAMCNEWS* article highlighted that many of these schools have developed ways to integrate such education into their curriculum [27]. For example, the University of Central Florida has implemented a module that educates students on topics related to pain management and opioid dependency and subsequently has students implement this new knowledge by developing treatment plans for patient cases that successfully prevent addiction development [27,28]. Another example is how Boston University, Harvard University, Tufts University, and University of Massachusetts medical schools now have a list of opioid-related competencies they must meet, which were developed by the governor of Boston [27,29]. Liaison Committee on Medical Education (LCME) reports show that 136/141 medical schools had curriculum content on substance abuse and pain management in 2015 [30].

While progress has certainly been made in adapting curricula to include opioid-related components, there is still more to be done. As the nature of the prescription opioid epidemic changes and heroin and fentanyl take a more central role, they should have more focus in related curriculum as well. While many schools have adapted curriculum to focus on opioid prescription and pain management, for schools that have not yet done so, curriculum should be further enhanced by adding more material and competencies related to the shift from prescription opioids to heroin and fentanyl, as well as how to treat such addictions in order to prevent future overdoses. Due to the alarming increase in heroin use and overdose, coupled with the transition from opioid misuse to heroin use and increased fentanyl added to heroin, a heightened focus on heroin education is needed to adequately prepare medical students for dealing with issues of heroin abuse, dependence, and overdose in the future.

## Medical education: opioids and heroin

The national scope of the opioid and heroin epidemic has reached a crisis among the healthcare community. In order to better prepare our future physicians, it is essential that they learn how to (1) identify patients at risk (2), recognize signs and symptoms of opioid and heroin abuse (3), follow proper opioid prescription guidelines, and (4) identify systems-based practice for referral of patients who are addicted. Therefore, when our graduates from medical schools are residents and future attending physicians, they will be prepared to effectively and efficiently respond to all situations, manage care, and prevent overdoses in the near future.

With incorporation of the opioid and heroin epidemic during medical education, the following critical aspect of curricula can be addressed: standard 7.2 organ systems/life cycle/primary care/prevention/wellness/symptoms/signs/differential diagnosis, treatment planning, impact of behavioral, and social factors to address the LCME Accreditation [31]. Medical schools can enhance current curriculum by integrating pertinent topics related to the rising heroin epidemic. For example, ongoing lectures throughout schooling can be given regarding patient populations that are prone to opioid and heroin use, information regarding the opioid to heroin transition, and ways to screen for opioid and heroin-seeking patients. It is necessary to educate students on signs and symptoms of overdose, as well as the proper response to overdose. This will include information about proper Naloxone administration. Students should also receive information regarding the role of fentanyl in heroin overdoses and be advised that reversing a fentanyl-related overdose requires Naloxone within a shorter amount of time and possibly additional doses [32].

Over prescription of opioids is one factor often cited as contributing to the rise of the opioid epidemic [33,34]. In response, there have been national and statewide changes in regulation guidelines for opioid prescribing [33]. Given the necessity of both prescribing pain medication and understanding how to do so safely, medical school curricula related to opioids should also include appropriate instruction regarding safe prescribing practices. This is not only important to prevent over prescription of opioids but also to prevent the growing concern of under prescribing pain medications as well [35]. A better understanding of current regulations and prescribing practices can minimize students’ hesitancy to prescribe opioids in the future and promote safe management of pain.

Given the gaps in physician expertise to treat substance abusing patients [23–25], it will benefit medical students to be exposed to different treatment methods for opioid and heroin abuse early in their careers. Application of this education can be achieved through simulation-based training with a focus on opioids and heroin.

## Simulation-based training: opioids and heroin

Over the past 20 years, simulation-based medical education (SBME) has become a frequently used asset in undergraduate medical education [36]. The benefits of SBME are well known, but most importantly, is its ability to provide medical students with repeatable clinical experiences that avoid patient harm [37]. Moreover, studies show that when training medical students on proper critical assessment and management of critically ill patients, students trained via an SBME method outperform students trained via a didactic lecture or problem-based learning method [38,39]. SBME has also been shown to enhance retention of training information compared to lectures [40]. While changing all aspects of medical curricula is important for preparing students to address the heroin and fentanyl crisis, simulation is a necessary component to ensure students are as best prepared as possible.

Creating scenarios for training through the use of SBME is expanding and may hold great advantages in training medical students in future interactions during the current national and international epidemic of heroin (and other opioids). This epidemic has become a Public Health Emergency of International Concern (PHEIC) and requires serious training, awareness, and engagement. SBME allows the use of partial task trainers, standardized patients, and virtual patients, all potentially incorporated into high-fidelity simulations [41]. SMBE involves cognitive and psychomotor skills that are not only confined to hand-eye coordination, the use of naloxone, securing airways, and cardiovascular resuscitation but may also be applied to social interactions with patients and their families. Instructors, or creators, of simulation scenarios must understand the topic/choice under examination that is intended for medical simulation and have a strategy for scenario development [42]. Such an approach that incorporates a psychosocial component, in effect, deploys the important concept of emotional intelligence (EI) in the interdiction of this PHEIC, where students and others learn to manage their emotions, those of others, and become acutely aware of the surroundings and environments in which they are interacting (and the consequences of their interactions) [43–45]. In this way, EI scenarios are created that assist learners in building confidence through skills and knowledge. Instructors should never overlook the fact that multidisciplinary approaches to scenario developments are extremely important [46]. Cooperation, collaboration, and interprofessionalism go hand in hand.

Students must become cognizant of facts pertaining to the individual patient, familial factors, occupational factors, and economic factors that are related to heroin addiction and relapse [47]. Such learning tasks can be effectively accomplished through SBME [48,49]. Simulation technology has reached the point where biomonitoring through galvanic skin sensors with heart rate reporting, electroencephalograms, and respiratory rates can be summarized in algorithms as levels of stress, cognitive workload, and learning [50]. This will assist with our ability to evaluate and assess learner knowledge and performance in various complex and stressful scenarios. The advantage of developing such technology is that biomonitoring and its relation to learning and cognitive load can now be accomplished through wearable wrist sensors. While this technological approach is very effective for the evaluation of learners in EI scenarios, the wearable wrist/digital technology is now being adopted and transitioned from the learner to the recovering addict leaving rehabilitation [51]. If consent is received from the individual, parties interested in the recovering addict’s wellbeing (family and health professionals) may monitor the individual’s physiological status (and even social inputs) with a built-in alerting ability linked to health professionals and emergency medical services (this is done through feed forward-back propagating neural networks where indicators can be converted into measurable outputs through development of a machine learning-based approach) [50]. Therefore, stress and learning, in addition to cognitive workload, can be assessed and applied, not only to the learner but also the recovering addict who has graduated from a rehabilitation program. In regard to the heroin PHEIC, SBME scenarios that make future physicians and allied health professionals aware of the predictors, situations, and reasons for relapse are extremely important and may impact patient, social, and economic outcomes [47,52–57].

## Conclusion

Physicians play an essential role in addressing the public health crisis of increased overdoses across the nation. Doctors have the ability based on their interactions with patients to potentially identify who may be at risk for or who is currently suffering from an opioid or heroin addiction. Unfortunately, many physicians feel ill-prepared and lack the knowledge and expertise to effectively identify, assess, and treat opioid and heroin addiction in their patients. Adapting medical school curricula is one essential way to address this knowledge gap. With the inclusion of topics related to opioid and heroin addiction and overdose in curriculum, medical schools have the potential to educate medical students early in their careers about the pressing opioid and heroin epidemic and adequately prepare students to identify and address issues of opioid and heroin abuse in their future careers. Moreover, using SBME can further enhance the training of medical students, providing them with simulated opportunities to develop and enhance both their skillset and EI, thereby enabling them to manage opioid and heroin addiction or overdose in future patient encounters. This is important for not only limiting the number of patients who are suffering from opioid and heroin addiction and improving patient outcomes but also to save lives by preventing overdose and relapse. Incorporating opioid and heroin addiction topics into medical school curriculum and SBME will enable medical schools to train our future physicians to be capable of preventing and recognizing opioid and heroin addiction, a crucial component in addressing the rising opioid and heroin epidemic across America.
